# The Ototoxicity of Antimalarial Drugs—A State of the Art Review

**DOI:** 10.3389/fneur.2021.661740

**Published:** 2021-04-20

**Authors:** Magdalena Jozefowicz-Korczynska, Anna Pajor, Weronika Lucas Grzelczyk

**Affiliations:** ^1^Balance Disorders Unit, Otolaryngology Department, The Norbert Barlicki Memorial Teaching Hospital, Medical University of Lodz, Lodz, Poland; ^2^Department of Otolaryngology, Head and Neck Oncology, The Norbert Barlicki Memorial Teaching Hospital, Medical University of Lodz, Lodz, Poland

**Keywords:** ototoxicity, antimalarials, dizziness, vertigo, tinnitus, hearing loss

## Abstract

This review summarizes current knowledge about the occurrence of hearing and balance disorders after antimalarial drugs treatment. It also examines the clinical applications of antimalarials, their mechanisms behind this ototoxicity and how it can be monitored. It includes studies with larger numbers of patients and those in which auditory function was assessed using audiological tests. Some antimalarials have been repurposed for other conditions like autoimmune disorders, rheumatic diseases, some viral diseases and cancers. While old antimalarial drugs, such as quinoline derivatives, are known to demonstrate ototoxicity, a number of new synthetic antimalarial agents particularly artemisinin derivatives, demonstrate unknown ototoxicity. Adverse audiovestibular effects vary depending on the medication itself, its dose and route of administration, as well as the drug combination, treated disease and individual predispositions of the patient. Dizziness was commonly reported, while vestibular symptoms, hearing loss and tinnitus were observed much less frequently, and most of these symptoms were reversible. As early identification of ototoxic hearing loss is critical to introducing possible alternative treatments with less ototoxic medications, therefore monitoring systems of those drugs ototoxic side effects are much needed.

## Introduction

Some medications have ototoxicity effects, that is, they influence the hearing (cochleotoxicity) and/or vestibular (vestibulotoxicity) end organs. The adverse effects (AEs) of ototoxic drugs may cause temporary or permanent hearing loss, tinnitus, dizziness, and vertigo, and may have neurotoxic effects on the auditory and balance systems. The best-known ototoxic drugs are aminoglycoside antibiotics, salicylates, non-steroidal anti-inflammatory drugs, loop diuretics, antimalarials (quinine) and platinum-based cytostatics. Ototoxicity is also reported in patients treated with immunosuppressants (cyclosporin, tacrolimus), antiviral drugs (pegylated and non-pegylated interferons, ribavirin, inhibitors of viral reverse transcriptase—HIV−1 antiretroviral), analgetics (acetaminophen, hydrocodone, methadone), and phosphodiesterase-5 inhibitors (1).

The online databases PubMed (Medline) and Embase (on OVID) were searched for articles published from 1985 to August 2020, with the most recent update on December 2020. The following MeSH terms and keywords were used: ototoxicity, antimalarial drug, antimalarial agent, vestibular symptom, dizziness, vertigo, tinnitus or hearing loss, adverse effect, drugs inducing ototoxicity. The search included publications written in English containing relevant data.

### Types of Antimalarial Drugs

Contemporary antimalarial drugs comprise several groups of medications:

- quinoline-type drugs (4-aminoquinolines: chloroquine, hydroxychloroquine, amodiaquine, pyronaridine, piperaquine; 8-aminoquinolines: primaquine, tafenoquine; aryl aminoalcohols: quinine, quinidine, mefloquine, halofantrine, lumefantrine);
- naphthoquinone (Atovaquone);
- antifolates (sulfadoxine-pyrimethamine);
- guanidine derived drugs (proguanil, cycloguanil, chlorproguanil);
- sesquiterpene lactones—artemisinin and its semi-synthetic derivatives (dihydroartemisinin, artemether, artesunate) currently used in artemisinin-based combination therapy (ACT);
- arterolane -a synthetic peroxide-containing compound (2–8).

The WHO recommends five types of ACT for use in children and adults as first and second-line treatment for uncomplicated malaria caused by infection with *Plasmodium falciparum*:

- artemether plus lumefantrine,
- artesunate plus amodiaquine,
- artesunate plus mefloquine,
- dihydroartemisinin plus piperaquine
- artesunate plus sulfadoxine-pyrimethamine (2).

Artemisinin-based combinations employ a short-acting artemisinin derivative in combination with other complementary compounds (long-acting or with different mechanisms of action) (4). WHO guidelines recommend the use of quinine plus clindamycin for pregnant women in their first trimester, and either ACT or chloroquine for the treatment of uncomplicated malaria caused by other species of *Plasmodium* (*P. vivax, P. ovale, P. malariae, P. knowlesi*); however, in case of chloroquine-resistant infections, ACT is used alone (5).

Chloroquine (CQ) and hydroxychloroquine (CHQ) are derivatives of quinine (QN), obtained from the bark of the Peruvian Cinchona tree. QN was first isolated in 1820 by the French scientists Pierre-Joseph Pelletier and Joseph-Bienaimé Caventou (9). CQ was first synthesized in 1934, and hydroxychloroquine sulfate (Plaquenil) was developed in 1946 as a less toxic chloroquine analog (10, 11). Another derivative is Mefloquine (Lariam), which was developed by the United States Army in the 1970s and came into use in the mid-1980s. It is commonly recommended as a prophylaxis for travelers to areas where malaria is endemic (12). Over the years drug-resistant strains of malaria have developed; the first example was found in the 1980s for QN, and since 2006, QN has no longer been recommended as a first-line treatment for malaria.

Artemisinin was discovered in 1972, and dihydroartemisinin in 1973, by Chinese pharmaceutical chemist Tu Youyou, who received the 2015 Nobel Prize in Physiology or Medicine for her discoveries concerning novel therapies against malaria (4). Similarly, Gertrude Elion and George Hitchings (4) also received a Nobel Prize in 1988 for their discovery of pyrimethamine. Some commonly-used antimalarial drugs discussed in the present review are presented in Table 1. Today, often for economic reasons, many antimalarial drugs are manufactured locally in endemic countries under different names (6–8).

**Table 1:** Commonly-used antimalarial drugs (2–4, 6–8).

**Drug**	**Trade name**	**Year of discovery/clinical use**	**Diseases/treatment**
Quinine	Qualaquin, Quinamm, Quiphile, Quinine sulfate, Cinkona	1820—isolation from bark of Chinchona tree 1944—chemical synthesis	Treatment of uncomplicated malaria in the first trimester of pregnancy (with clindamycin); Treatment of severe malaria when artemisinins are not available
Chloroquine	Aralen, Arechine, Arequin, Chingamin, Resochin, Dawaquin, Lariago, Nivaquine	1934, 1947 in clinics	Treatment of uncomplicated malaria (*P. vivax, P. ovale, and P. malariae*, susceptible strains of *P. falciparum)*; Rheumatic and autoimmune diseases; Viral diseases (Covid 19); Extraintestinal amebiasis
Hydroxychloroquine	Plaquenil, Hydroquin, Dolquine, Quensyl, Quinoric, Plaquinol, Axemal	1946 1955—approval for medical use in the US	Treatment of uncomplicated malaria due *to P. falciparum, P. malariae, P. ovale, P. vivax*; Prophylaxis of malaria in areas where chloroquine resistance is not reported Discoid and systemic lupus erythematosus and rheumatoid arthritis in adults
Mefloquine	Lariam, Mephaquin, Mefliam, Larimef	mid-1970s (US)	Prophylaxis of malaria (all species)
Halofantrine	Halfan	1965 to 1975	Treatment of malaria (severe and resistant forms)
Amodiaquine	Basoquin, Camoquin, Amdiaquine, Amobin	1948	Treatment of uncomplicated malaria;
Primaquine	Primaquine, Primac, Evaquin, Malirid	1946 1952—approval in the US	Radical cure (prevention of relapse) of *P.vivax and P. ovale* malaria^*^; Treatment (with clindamycin) of *Pneumocystis Jirovecii* pneumonia
Tafenoquine	Arakoda, Krintafel Kozenis, Kodatef	1978 2018—FDA approval for antirelapse therapy (Krintafel) and for chemoprophylaxis(Arakoda)	Radical cure (prevention of relapse) by P. vivax malaria^*^; Prophylaxis of malaria in patients aged 18 years and older
Pyrimethamine	Daraprim	1952	Toxoplasmosis; Prevention *Pneumocystis jiroveci* pneumonia in HIV/AIDS; Malaria (with sulfadoxine)
Artemether	Larither, Rapither	1975 (China)	Alternative for treatment of severe malaria when artesunate is not available
Artesunate	Artesun, Larinate	1977 2020—approval for medical use in US	Initial treatment of severe malaria in adults and children (including infants, pregnant and lactating women)
Atovaquone	Mepron	1986 (China)	Prevention and treatment mild to moderate of *Pneumocystis Jirovecii* pneumonia Toxoplasmosis;babesiosis; Malaria (with proguanil)
Proguanil	Paludrine	1945	Prevention and treatment of chlorochine -resistant *P.falciparum* malaria
**Combination therapies**			
Atovaquone + proguanil (A + P)	Malarone, Malanil	2000s	Prophylaxis of malaria in travelers; Treatment of uncomplicated *P. falciparum* malaria;
Sulfadoxine-pyrimethamine (SP)	Fansidar	1981—approval for medical use in the US	Treatment of acute, uncomplicated *P*. *falciparum* malaria in patients with chloroquine resistance; Intermittent preventive treatment of malaria in pregnancy
Artemether + lumefantrine (A-L)	Coartem, Riamet, Falcynate-LF Laritem/Lumerax	1985 (China), 1992—medical use	Treatment of uncomplicated malaria (*P.falciparum, P.vivax*, considered effective against *P. knowlesi, P. ovale, P. malariae*)
Artesunate (AS) + amodiaquine (AQ)	Camoquin plus, Coarsucam/ASAQWinthrop	2007	Treatment of uncomplicated *P.falciparum* and *P.vivax* malaria
Artesunate (AS) + mefloquine (MQ)	Artequin, ASMQ, Mefliam Plus, Falcigo Plus	1990s	Treatment of uncomplicated *P.falciparum* malaria
Dihydroartemisinin (DHA) + piperaquine (PPQ)	Eurartesim, DuoCotecxin, Artekin, Malacur, Ridmal	2011—approval for medical use in Europe	Treatment of uncomplicated *P.falciparum* and *P.vivax* malaria, likely to be effective against *P. knowlesi, P. ovale, P. malariae*
Artesunate (AS) + sulfadoxine-pyrimethamine (SP)	Amalar plus, Co-arinate FDC, Falciart Kit, Larinate Kit, Malosunate		Treatment of uncomplicated *P.falciparum* malaria
Artesunate (AS)+ pyronaridine	Pyramax	2017—WHO recommendation	Treatment of uncomplicated *P.falciparum* and *P.vivax* malaria
Amodiaquine (AQ) + sulfadoxine-pyrimethamine (SP)	SPAQ-CO	2012—WHO recommendation	Seasonal malaria preventative use in young children

* *Radical cure—using a drug to target the hypnozoite (dormant stage of the parasite in the liver) in combination with standard anti-malarial drugs (such as chloroquine or ACTs), so blood and liver stages of P.vivax are eliminated*.

### Clinical Applications of Antimalarial Drugs

Antimalarials are used not only for the treatment of malaria but also for a range other dermatological, immunological, rheumatological, and severe infectious diseases. In the past, quinine has been used to treat otologic conditions (Menière's disease, herpes zoster, vertigo, purulent otitis media, furuncles in the auditory canal) due to its analgesic as well as ototoxic properties (9). Nowadays, chloroquine and hydroxychloroquine are used to treat autoimmune and connective tissue diseases like systemic lupus erythematosus (SLE), rheumatoid arthritis, Sjögren's syndrome, sarcoidosis, palindromic rheumatism, eosinophilic fasciitis, dermatomyositis, cellulitis, mixed and undifferentiated connective tissue disease (11, 13, 14). They have also demonstrated anticancer properties against different types of cancer (e.g., colon, lung and breast cancer, central nervous system tumors, hematological malignancies), and against cancer cell lines *in vitro* (11, 15). CQ has also demonstrated antiviral activity, and has been used in clinical trials in infections caused by hepatitis C virus, dengue virus-2, human immunodeficiency virus, and Chikungunya virus (15). Recently, CQ and CHQ were proposed as treatments for severe acute respiratory syndrome coronavirus-2 (SARS-CoV-2) infection and were prescribed for almost 12% of COVID-19 patients in Europe (16). The antimalarials have also been used to treat metabolic diseases. Chloroquine shows antihypertensive, antilipidemic, and hypoglycemic effects (11), while artemisinin and its derivatives have been used in the treatment of type 2 diabetes mellitus (17). Currently, CQ and CHQ are being tested for neurological diseases such as neurosarcoidosis, as well as chronic lymphocytic inflammation with pontine perivascular enhancement responsive to corticosteroids, and primary progressive multiple sclerosis (13).

## Mechanisms of Ototoxicity

Although they have been studied in animal models, particularly quinine (18), the mechanisms underlying the ototoxicity of antimalarials remain poorly understood. Quinine can negatively affect the auditory system at the level of the central auditory pathway and the auditory periphery. Several hypotheses about changes in cochlea have also been proposed, such as impairment of outer hair cells (OHCs), reduction in the blood flow, and microangiopathy due to quinine-induced thrombocytopenia and disseminated intravascular coagulation (19–21).

Quinine-induced morphological and physiological changes have been observed in the cochlea: in guinea pigs, ultrastructure lesions have been observed, including swellings of the subsurface cisternae and the formation of a central microtubule core in OHCs (22), and high doses of quinine have been found to cause elongation of isolated OHCs followed by contraction (23). Exposure to 5.0 mM QN had the same effect of elongation, but without subsequent relaxation of isolated guinea pig cochlear OHCs, and appeared to reduce active force generation in OHCs without any effect on compliance (24). Recently Davis et al. demonstrated that CQ and CHQ treatment causes damage to hair cells in the zebrafish lateral line while chloroquine causes dose-dependent loss of outer hair cells in cultured neonatal mouse cochlea, with more loss observed in the basal turn than the apical turn, without reduction of supporting cells (25).

In an isolated temporal bone preparation, QN treatment altered the mechanical response of the basilar membrane induced by sound (26). Zheng et al. demonstrated that QN decreased the mean amplitude of the electrically-evoked otoacoustic emissions (OAE), affecting *in vivo* electromotility of OHCs (27). Dieler et al. report that QN changes the membrane potential of hair cells (hyperpolarization, then depolarization) in a dose-dependent and reversible manner, and causes a diminution of evoked rapid motile responses without any alterations in the turgor, shape or fine structure of OHCs (28). Perilymph perfusion with quinine in guinea pigs was found to result in a reduction of cochlear microphonics and summating potential (at 100 pmol/L), as well as compound action potential input-output function (at all intensities), but no change in endocochlear potential (29). The authors conclude that this lack of change in endocochlear potential excludes the stria vascularis as QN site of action and suggest hearing loss may occur through inhibition of the Ca++-activated K+ channel or an ATP-sensitive K+ channel.

In guinea pigs injected with quinine, the amplitudes of auditory brainstem response (ABR) were reduced at all sound levels but less so at high levels, while distortion product otoacoustic emissions (DPOAE) amplitudes were unchanged at high stimulus levels (30). This could suggest that QN affects both outer and inner hair cells (IHCs) as well as spiral ganglion neurons (SGNs). It has been demonstrated that QN also affects other auditory sites, like the spiral ganglion, and auditory neurons. In adult isolated mice spiral ganglion neurons (SGNs), QN was found to reversibly reduce amplitude and prolong the duration of action potentials; it also blocked the whole-cell potassium and sodium currents, but not calcium currents (31). Quinine modulated the auditory cortex in cats in a different way: it reduced the spontaneous firing rates in the primary auditory cortex and anterior auditory field while increasing them in the secondary auditory cortex (32). Artemisinin derivates (dihydroqinghaosu, artemether, and arteether) may cause necrosis of specific brain stem nuclei in animals, particularly those involved in hearing and balance (33, 34).

There are also few reports about the ototoxicity of other antimalarials. Mefloquine caused dose-dependent damage to hair cells (starting from the cochlear base), supporting cells (prior to hair cells), and SGNs in cultures from postnatal developing rats (35, 36). This mechanism may be due to different apoptotic pathways (the death receptor-mediated signaling pathway or the mitochondrial pathway), as the expression of numerous pro, and anti-apoptotic genes were reported in the cochlear epithelium and SGNs in rat (36, 37). MQ increased oxidative stress in the cochlear hair cells and SGNs, it also induced caspases-3-mediated apoptosis; it is believed that the coenzyme nicotinamide adenine dinucleotide (NAD+) plays a protective role in this process (38). Mefloquine also induced dose-dependent vestibular hair cell loss in the utricle in postnatal rat and the process has been attributed to activation of apoptosis by caspase-8 and caspase-9 (39). Halofantrine affected various cochlear structures, such as IHC, OHCs, SGNs and phalangeal cells in guinea pigs (40). QN also induced tinnitus in the behavioral model of tinnitus in rats (41); however, in another study animals treated with 200 mg/kg/d QN did not exhibit tinnitus-like behavior when assessed using the gap prepulse inhibition of acoustic startle (42).

Interestingly, quinoline drugs have been found to prevent ototoxicity by loop diuretics (furosemide/ethacrynic acid), aminoglycosides, and cisplatin in animal models based on chinchilla and zebrafish (20, 43–45). QN derivatives reduce uptake of gentamicin, neomycin, and cisplatin into hair cells (44, 45). Although the molecular mechanism is unknown, quinine probably acts as a blocker of mechanotransducer (MET) channels for gentamicin (20, 46); however, chloroquine does not appear to reduce MET channel activity (25).

## Clinical Manifestations of Ototoxicity

### Cochleotoxicity—Hearing Loss and Tinnitus

Antimalarial drugs, such as CQ and HCQ, were considered for a long time as having audio-vestibular side effects (47, 48). QN overdose is generally known as cinchonism. However, audiovestibular toxicity is not a common adverse effect of antimalarials: only 61(2.6%) such complications were reported for all 2339 antimalarial adverse effects listed in the French Pharmacovigilance Network database between January 1986 and December 2010. These complications were nearly equally distributed between CQ and HCQ, 50.8% after therapy for autoimmune diseases and 26.2% in malaria (49). Quinine was not found to have a significant effect on the risk of hearing loss in a study of ototoxic medication use and the 10-year cumulative incidence and progression of hearing loss in 3753 older adults (50). However, this may be due to the low proportion of people using these medications (1.1%) or the fact that they were used for a brief period. In a large group of 19,850 patients who received different regimens containing mefloquine (alone or in combination with artesunate, artemether or sulfadoxine-pyrimethamine) due to uncomplicated malaria, the frequency of dizziness was 47.2% and hearing loss 3.44%; after 28 days of follow-up, these values fell to 6.49 and 0.52% respectively (51).

Hearing loss is typically bilateral, mild to moderate sensorineural and mostly reversible (10). Many case reports have described ototoxic effects after treatment with antimalarials, especially CQ and HCQ (52); however, this review focuses on studies based on larger groups of patients. Studies reporting an association between antimalarial use and cochlear ototoxicity are summarized in Table 2, whereas those not finding any such association are listed in Table 3.

**Table 2:** Drugs associated with cochlear ototoxicity.

**Drug**	**Diagnosis**	**No patients**	**Study design**	**Method**	**Results of hearing tests**	**Tinnitus**	**References**
Chloroquine Phosphate	Malaria	*n*-30 (14–58 yrs)	Short term chloroquine-induced hearing loss	ABR OAE	6.7% SNHL, ab-ABR and OAE, after 1 mo all normal	ND	Subramaniam and Vaswani (53)
Atovaquone-proguanil (A+P) Artemether-lumefantrine (AM-L) Quinine sulfate (QN)	*P. falciparum* malaria	*n*- 97 (6–50 yrs)	A+P vs. AM-L vs. QN	PTA OAE ABR	PTA and DPOAE transient significant SNHL in patients treated with quinine	ND	Gürkov et al. (54)
Artesunate-amodiaquine (AS+AQ) Artemether-lumefantrine (AM-L)	Uncomplicated malaria	*n* −116 (AS+AQ) *n* −111 (AM-L) (6 mo to 14 yrs)	AS + AQ vs. AM-L	PTA	No differences of subjects and controls after 9 to 12 mos	ND	Adjei et al. (55)
Artesunate- mefloquine (AS+MQ)	*P. falciparum* malaria	*n* −93 (13–53 yrs)	AS + MQ	PTA TYMP ABR	57% SNHL associated with age only	ND	Carrara et al. (56)
Artemether-lumefantrine (AM-L)	Uncomplicated *P.falciparum* malaria	*n* −150 (19–65 yrs) control *n*- 150	AM + L vs. control	PTA	Subjects receiving AM-L had significantly greater SNHL hearing loss	ND	Toovey and Jamieson (57)
Quinine-dihydrochloride (QN)	*P. falciparum* malaria	*n* −9 (20–49 yrs) control *n*-12	QN vs. control	PTA	All pts with *P. falciparum* had unilateral SNHL, after 1 week all normal	YES	Tange et al., (58)

*SLE, systemic lupus erythematosus; SNHL, sensorineural hearing loss; PTA, pure-tone audiometry; OAE, otoacoustic emission; ABR, auditory brainstem response; TYMP, Tympanometry; ND, no data; mo, month*.

**Table 3:** Drugs not associated with cochlear ototoxicity.

**Drug**	**Diagnosis**	**No of patients**	**Study design**	**Method**	**Hearingresults**	**Tinnitus**	**References**
Artemisinin-naphthoquine (ART-NQ)	Malaria	*n* −48 (5–12 yrs)	ART-NQ vs. control in children	PTA, whispered voice test, Rinne's, Weber's tests	No sign of ototoxicity	ND	Benjamin et al. (59)
Artemether-lumefantrine (AM-L) Atovaquone-proguanil (A+P) Artesunate-mefloquine (AS+MQ)	Uncomplicated *P. falciparum* malaria	*n* −159 (AM-L) *n* −53 (A+P) *n* −53 (AS+MQ) (12–56 yrs)	AM-L vs. A+P vs. AS+MQ	PTA TYMP ABR	In all three groups, small improvements (2–4 dB) in PTA	ND	Carrasquilla et al. (60)
Artesunate- amodiaquine (AS+AQ)Artemether-lumefantrine (AM-L)	Malaria	*n* −184 (AS-AQ) (11.94 ± 10.52) *n* −182 (LA) (11.75 ± 9.17)	AS + AQ vs. fixed-dose AM-L	PTA	No sign of ototoxicity	ND	Ndiaye et al. (61)
Artemether-lumefantrine (AM-L)	Malaria	*n* −68 (7–65 yrs) Control *n*−68	AM-L vs. control	PTA TYMP ABR	No differences in results cases vs. controls	ND	Hutagalung et al. (62)
Chloroquine (CHQ)	SLE	*n* −9 (7.6+4.4 yrs)	Children SLE mother exposed during pregnancy vs not exposed to CHQ	PTA	Lack of permanent fetal ototoxicity in children exposed to CHQ	ND	Borba et al. (63)
Artemisinin or artesunate Artemisinin-mefloquine (ART+MQ) Mefloquine (MQ)	Malaria	*n* −350 (4–65 yrs) control *n*-180	Antimalarial drug vs. control	PTA ABR	No differences between the groups	ND	Kissinger et al. (64)
Artemisinin/artesunate	Malaria	*n* −79 (3–53 yrs) control *n*-79	Artemisinin/artesunate vs. control	PTA ABR	No sign of ototoxicity	ND	Van Vugt et al. (65)

*SLE, systemic lupus erythematosus; SNHL, sensorineural hearing loss; PTA, pure-tone audiometry; OAE, otoacoustic emission; ABR, auditory brainstem response; TYMP, tympanometry; ND, no data*.

The methods used to assess auditory function in the studies varied from subjective to objective audiometry reports. The most common technique was pure tone audiometry, with OAE and ABR being used in children. Most studies used a single hearing test method; however some used three or more. Audiotoxicity manifestations vary depending on the antimalarial drugs. The effects of long-term use of conventional antimalarial drugs like QN have been adequately established. In a study where quinine-dihydrochloride was administered intravenously to patients with malaria caused by *P. falciparum*, all patients reported severe impaired hearing loss, tinnitus, or a feeling of pressure on the ears. Twenty-four hours later or 1 week later, all audiograms had returned to normal, and the adverse effects disappeared (58). In another study, artemether+lumefantrine (AM-L), atovaquone+proguanil (A+P) and quinine sulfate were compared using a complete audiometric evaluation (PTA, OAE, ABR); it was found that only the patients who were treated with quinine demonstrated a significant but transient SNHL (54). HCQ ototoxicity is less common. Subramaniam and Vaswani found ototoxicity to be infrequent and reversible when chloroquine is administered in regular doses to treat uncomplicated malaria (53).

Artemisinin-based combination therapy (ACT) has contributed remarkably to decrease malaria illnesses and deaths; however, ACTs may affect the auditory system (5, 66). Artesunate+amodiaquine (AS+AQ) and artemether+lumefantrine (AM-L) are two ACT regimens that have been extensively established for the treatment of uncomplicated malaria in Africa (55). Subjects receiving AM-L combination therapy have been found to have a significantly greater risk of SNHL (57). On the other hand, no differences in audiological results were found between a group of 68 patients treated for malaria and control subjects that had never received AM-L from the same endemic malaria region of the Myanmar-Thailand border (62). In addition, hearing loss was found to be common on admission (57%) and only associated with age in patients treated for 3 days with AS+MQ (56). Ndiaye et al. report no signs of ototoxicity in AS+AQ or fixed-dose AM-L (61). In another study, no differences in ABR and PTA were found between patients treated with artemisan or artesunate alone, artesunate + MQ and MQ (64); in addition, no audiometric differences were found between patients treated for acute malaria with an artemisinin derivate and a control group that had never received it (65). A randomized study by Carrasquilla et al. found different combinations of ACT yielded improvements in PTA in acute uncomplicated *P. falciparum* malaria (60). In addition, a single-dose of artemisinin-naphthoquinone (ART-NQ) was found to be well-tolerated and safe for treating uncomplicated pediatric malaria in patients with *P. falciparum* and *P. vivax* (59); in addition, exposure to chloroquine diphosphate during gestation was also found not to result in hearing impairment in children (63).

### The Adverse Effects of Antimalarials on the Vestibular System

Antimalarial drugs can affect the central and peripheral vestibular systems. However, vestibular ototoxicity related to the peripheral system, such as vertigo, nystagmus and imbalance, is rarely reported either in patients being treated for malaria or autoimmune disease, or in healthy persons taking the drugs for malaria prophylaxis, such as travelers. The peripheral vestibular system can result in either partial or complete impairment of the vestibular end organs. In 2012, lasting vestibular disorder was added to the list of adverse effects of mefloquine by the discovery that vertigo and loss of balance could be permanent in some cases (67).

Only a few authors have reported incidents of acute vertigo in patients treated for malaria. Subramaniam and Vaswani assessed short-term chloroquine-induced ototoxicity in 30 patients with malaria, including only one patient with vestibular adverse effects (53). That patient had vertigo and spontaneous nystagmus, which resolved on completion of therapy. Vestibulopathy in a woman with a history of Sjögren's syndrome was described as a result of an accidental CHQ overdose. The patient had some neurological and otological complaints, such as bilateral tinnitus, imbalance, postural disequilibrium, and vertigo. After 12 weeks, only a mild improvement of symptoms was observed (68). The most critical causes for discontinuation of MQ prophylaxis among travelers are ototoxic and neuropsychiatric adverse effects. The most typical adverse effects in malaria prophylaxis are headaches (15.5%) and dizziness (14.4%), with vertigo and visual difficulties reported in 1–10% of prophylactic users (69). In addition, a meta-analysis of 35 cohort studies (198,493 participants) comparing prophylactic MQ intake with placebo in adults, children and pregnant women indicated that MQ users were more likely to report nausea (high-certainty evidence) and dizziness (high-certainty evidence) (12).

However, a clinical–pharmacological study with 22 healthy volunteers, identified vertigo (96%), nausea (82%), and headache (73%) after administration of MQ (750 and 500 mg at an interval of 6 h), during 21 days of monitoring. In 73% of subjects, severe vertigo was reported, which required bed rest and medication for 1 to 4 days. The majority (77.3%) of the participants showed symptom resolution within 3 weeks after drug administration. The authors emphasize that the severity of adverse effects after the usual therapeutic dosage of MQ in healthy subjects were unexpectedly high and should be taken into account during the treatment of malaria in travelers (70).

Central vestibulopathy was reported in a previously healthy 24-year-old male, taking three doses of MQ for prophylaxis malaria in Africa, despite evolving and worsening symptoms. He developed prolonged psychosis, anxiety, paranoia, short-term memory impairment, personality change and disequilibrium, vertigo, and tinnitus. He had downbeat nystagmus during normal routine vestibular responses in videonystagmography. Vestibular-evoked myogenic potentials on the right-side were enhanced as compared to the left side. Computerized dynamic posturography revealed a dysfunction pattern with falls during sensory organization tests (SOT) 5 and 6, so somatoform disorder was suspected. Radiological imaging tests showed insignificant changes. The author speculates that the symptoms were caused by damage or imbalance between the oculomotor and vestibular nuclei due to multifocal injury to the drug-induced brainstem. In 10 months follow-up, some improvement was observed, but still, he had episodes of vertigo and disequilibrium (71).

To improve efficacy, prevent transmission and reduce resistance, numerous antimalarial drug combinations and newer drugs have been introduced. However, such combinations may increase the level of adverse effects through cumulative toxicity. Similar incidences of adverse effects were observed between groups of travelers receiving atovaquone-proguanil A-P (493 subjects) or MQ (483 subjects) for the prevention of *P. falciparum*–induced malaria, that is, 71.4% in A-P vs. 67.3% in MQ group. The respective incidences of dizziness and vertigo were found to be 2 and 9%. The authors stated that A-P was better tolerated than MQ, and it was similarly effective for malaria prophylaxis in non-immune travelers (72).

### Dizziness—A Sign of Neurotoxicity of the Balance System

The Barany Society Classification of Vestibular Disorders defines dizziness as a non-vertiginous sensation of disturbed or impaired spatial orientation without a false or distorted sense of motion (73). The Barany Society suggests that dizziness and vertigo should be defined separately. Subjects complaining of dizziness describe a range of sensations, such as feeling faint, weak or unsteady. In clinical studies, dizziness is classified as neurological manifestation (69). Headaches (15%) and dizziness (14%) are the most common mental and neurological adverse effects of antimalarial drugs during the treatment of acute malaria or prophylaxis (69).

Among antimalarial drugs, MQ is particularly frequently analyzed because it affects more domains of mental and neurological manifestations. For example, among 5,332 reactions associated with MQ used in malaria prophylaxis, Nevin and Leoutsakos reported vertigo in 6.3% of cases and dizziness in 17.3% (74). Potasman et al. assessed neuropsychiatric problems in 2,500 young travelers to tropical countries who received malaria prophylaxis (75). The most common were sleeping disturbances (52.1%), fatigue (48.7%), and dizziness (39.3%). A study of 1,170 Swedish soldiers who returned from Liberia and used MQ or atovaquone-proguanil (A-P) as malaria chemoprophylaxis found dizziness to be reported by 10.6% of MQ group and by 3.7% of A-P group (76).

Some studies have examined the adverse effects associated with combinations of malaria drugs. Lula et al. analyzed the safety and tolerability of artesunate and amodiaquine combination in the treatment of 387 patients with uncomplicated malaria (77). Dizziness was observed in 16.9% of cases, hypoacusis in 0.3%, tinnitus in 1.6%, and blurred vision in 1.6%. Vugt et al. compared effectiveness of artemether-benflumetol (AB, 177 patients) and artesunate-mefloquine (AM, 232 patients) in the treatment of *P. falciparum* malaria (78). Significantly higher rates of dizziness were found in the AM group (35%) than the AB group (15%). In addition, Odur et al. (79) reported dizziness in 18.4% of 299 malaria patients treated with dihydroartemisinin-piperaquine; this was the second most frequent adverse effect after weakness (32.6%) (77). Vestibular adverse effects are summarized in Table 4.

**Table 4:** Drugs associated with vestibular ototoxicity.

**Drug**	**Diagnosis**	**No patients**	**Vestibular and central neurological signs**	**References**
Chloroquine phosphate	Malaria	*n* −30 (14–58 yrs)	1 pt vertigo, nystagmus—resolved on completion of therapy	Subramaniam and Vaswani (53)
Accidental hydroxychloroquine overdose	Sjögren	64-year-old woman	Bilateral tinnitus, imbalance, ataxia, vertigo Postural disequilibrium	Chansky and Werth (68)
Atovaquone-proguanil (A+P) vs. Mefloquine (MQ)	Travelers	A-P (*n* −493) MQ (*n* −483)	Dizziness vertigo A-P 2 vs. MQ 9% Neuropsychiatric AEs A-P 14 vs. MQ 29%	Overboschet al., (72)
**Prophylaxis**				
Mefloquine	Healthy volunteers	*n* −22 (27–4.5 yrs)	96% vertigo—symptom resolution within 3 weeks	Rendi-Wagner et al. (70)
Mefloquine	Previously healthy	24-year-old male	Disequilibrium, vertigo, tinnitus, downbeat nystagmus; Diagnosis - central vestibulopathy	Nevin (67)
Mefloquine with placebo	Short—long term travelers	198,493 participants 35 cohort studies	Nausea (high-certainty evidence) and dizziness (high-certainty evidence)	Tickell-Painter et al. (12)

*AEs, adverse effects*.

## Assessment and Monitoring of Ototoxicity

### Audiological Monitoring of Ototoxicity

Early identification of ototoxic hearing loss is critical to introducing possible alternative treatments with less ototoxic medications. However, hearing impairment may be unavoidable in some cases, even with ototoxicity monitoring. It is also necessary to bear in mind that some patients are predisposed to ototoxicity (19). One protocol for cochleotoxicity monitoring comprises baseline testing with conventional pure tone audiometry (PTA), high-frequency audiometry (HFA), tympanometry, speech audiometry, OAEs, and ABR. If the PTA could not identify initial ototoxic damage, objective audiological tests are important (80).

### Otoacoustic Emissions

OAE tests provide an objective evaluation of subclinical changes in cochlear OHCs (81) and are sensitive tests for detecting and monitoring even early small impairments in the inner ear due to ototoxicity. OAE measurement in children is a particularly attractive approach as an efficient objective test. Ototoxicity monitoring found simultaneous decreases in OAE responses and changes in high-frequency audiometry thresholds (82). However, one consideration in OAE testing is that most applications can produce errors at high frequencies and can be problematic in patients with hearing loss. OAEs do not require a behavioral response and are time efficient. Many significant criteria have been proposed to interpret OAEs, but none has gained universal acceptance so far (80).

### High-Frequency Audiometry

HFA comprises air-conduction threshold testing for frequencies above 8000 Hz, ranging up to 16 or 20 kHz. Nowadays, HFA is a well-established and widely-used method in ototoxicity monitoring programs; however, it is not standardized due to the fast decline of high frequencies with age and large inter-subject variability. HFA is more sensitive to early ototoxic changes than conventional audiometry and DPOAEs (19, 80).

### Auditory Brainstem Response

The ABR is generated by eight cranial nerve and auditory brainstem structures in response to sound stimuli presented to the ear. It has several clinical applications, including the diagnosis and monitoring of dysfunction in the eight nerves and auditory brainstem, and can be used to estimate the auditory threshold particular in the pediatric population. The ABR can reflect changes in amplitude and/or latency of neural responses as a result of ototoxicity (83). Compared to OAEs, ABR responses can be recorded in ears with more severe pre-existing hearing loss. ABR can be used in cases with moderate hearing loss but output restraint at high frequencies. The limitation of this procedure is its long duration.

### Tinnitus Evaluation

No formal tinnitus ototoxicity monitoring procedures have been designed, perhaps because tinnitus is not frequently mentioned as a side effect of antimalarial drugs (58, 68, 71, 77, 78). In the few existing guidelines on ototoxicity, tinnitus is mainly analyzed based on patient self-reporting (18, 19, 80). Konrad-Martin et al. suggest the use of the Tinnitus Ototoxicity Monitoring Interview to detect the onset of tinnitus and its changes in patients (84).

### Vestibulotoxicity Monitoring

The vestibular toxicity of some drugs is well-established and can vary from discreet to severe instability, caused by a total bilateral loss of vestibular function. The degree mostly depends on the extent of cellular damage within the vestibular end-organ (85). No widely accepted guidelines for vestibulotoxicity monitoring exist. Dizziness can be assessed using patient self-reporting questionnaire like the Dizziness Handicap Inventory (DHI) (86); patients with a greater DHI score have more extensive functional impairment, as confirmed by clinical tests. When systematically monitoring patients for ototoxicity, DHI questioning is strongly encouraged (80). Electronystagmography (ENG) or videonystagmography (VNG) with rotation chair testing are recommended, and the caloric test is highly sensitive to the presence of peripheral vestibular system impairment. In addition, quantitative techniques like vestibular evoked myogenic potentials (VEMPs) and computerized dynamic posturography may be used (19, 80). Vestibulotoxicity monitoring is essential for patients demonstrating signs of balance disorders in the course of treatment. On the other hand, some clinical vestibular tests like VNG or VEMPs would be impractical, and are not recommended for routine monitoring in the acute phase of the disease as they are poorly tolerated. There is a need to monitor vestibular system function before, during, and after medical therapies that employ drugs with ototoxic properties to allow a possibility to intervene (i.e., by reducing dosages or substituting less damaging medications) to stave-off permanent damage.

## Final Remarks

It is challenging to assess ototoxicity in clinical studies because the audiovestibular impairment in diseases treated by antimalarials, such as malaria, SLE or COVID-19, may also be related to the disease itself (87–89). In humans, hearing loss may also be caused by age, adverse effects of other drugs given simultaneously, or the development of concomitant diseases. In malaria, the disease may be affected by the endemicity status of malaria, and individual factors like malaria resistance, which may vary across the eligible studies (69). A 4-year follow-up study found 31 children to show a decrease in transitory evoked otoacoustic emissions (TEOAEs), suggesting that cochlear malfunction perseveres after recuperation from severe malaria (90).

Although for many years, ototoxicity in malaria patients was attributed to antimalarial drugs, studies on animal models suggest that the course of malaria might involve the inner ear. Significant hearing impairment in mice with cerebral malaria was confirmed; however, temporal bone examination revealed no structural alterations in the cochlea or any malaria-typical vascular lesions like leukocyte sequestration or micro-hemorrhages (91, 92). Schmutzhard et al. report an increase of intercellular adhesion molecule-1 (ICAM 1) expression in marginal cells of the stria vascularis in infected mice compared to control; however, there were no differences between malaria mice with and without hearing loss (85). They suggest that the endocochlear potential in the stria vascularis may be altered due to malfunction of the type 1 fibrocytes in the spiral ligament and disruption of the blood labyrinth barrier, which may cause the hearing loss observed in murine cerebral malaria (92, 93).

This review summarizes current knowledge about the occurrence of audiovestibular disorders after antimalarial drug treatment. Antimalarials are widely used not only to treat and prevent malaria but some have been repurposed for treating many autoimmune and rheumatological diseases, viral diseases, particularly SARS-CoV-2 infection, and various types of cancer. The ototoxic manifestations of antimalarial drugs include dizziness and vestibular symptoms; hearing loss and tinnitus were observed much less frequently and most of these symptoms were reversible. Early identification of ototoxic hearing loss is critical for introducing possible alternative treatments with less ototoxic medications. Therefore, more effective monitoring systems are needed.

## Author Contributions

All authors listed have made a substantial, direct and intellectual contribution to the work, and approved it for publication.

## Conflict of Interest

The authors declare that the research was conducted in the absence of any commercial or financial relationships that could be construed as a potential conflict of interest.

## Abbreviations

ABR: auditory brainstem response
ACT: artemisinin-based combination therapy
AEs: adverse events
CQ: chloroquine
CHQ: hydroxychloroquine
DPOAE: distortion product otoacoustic emissions
IHCs: inner hair cells
MQ: mefloquine
OAE: otoacoustic emissions
OHCs: outer hair cells
*P*: *plasmodium*
PTA: pure tone audiometry
QN: quinine
SGNs: spiral ganglion neurons
SLE: systemic lupus erythematosus
SNHL: sensorineural hearing loss.
